# Supplementary material for: FRCaMP, a Red Fluorescent Genetically Encoded Calcium Indicator Based on Calmodulin from Schizosaccharomyces Pombe Fungus
Source: Int J Mol Sci. 2020 Dec 24;22(1):111. doi: 10.3390/ijms22010111 (PMC7794825; doi:10.3390/ijms22010111)
Supplement: Supplementary file 1 [file ijms-22-00111-s001.pdf]

Supplementary Information

FRCaMP, Red Fluorescent Genetically Encoded Calcium Indicator Based on Calmodulin from Schizosaccharomyces Pombe Fungus

Oksana M. Subach, Natalia V. Barykina, Elizaveta S. Chefanova, Anna V. Vlaskina, Vladimir P. Sotskov , Olga I. Ivashkina, Konstantin V. Anokhin, and Fedor V. Subach

Supplementary Tables and Figures

**Table S1**	**2**
**Table S2**	**3**
**Figure S1**	**4**
**Figure S2**	**5**
**Figure S3**	**6**
**Figure S4**	**7**
**Figure S5**	**8**
**Figure S6**	**9**
**Figure S7**	**10**

Table S1. List of primers.

Primer	Primer sequence (5′-3′)
**RSP-BglII**	CTGAGATCTATGAGGAAACCGTTCCGTG
**CaMSP-HindIII-r**	CCAAGCTTCTACTTGGAAGAAATGACACGAGAGAATTC
**MSP-3-5**	ggtAGATCTatgAGAAAAnnsnnsnnsACTGCCTATAACGCTGTAC
**MSP-6-8**	ggtAGATCTatgAGAAAAACGTTCCGTnnsnnsnnsAACGCTGTACGTGCTTTC
**MSP-9-11**	ggtAGATCTatgAGAAAAACGTTCCGTACTGCCTATnnsnnsnnsCGTGCTTTC AACACTTGG
**dRSP-BglII2**	cgagatctGTGCCGCGGGTTTCCGAG
**BamHI-bJun**	TGGggatccgccaccatggTGAAGGCGGAGAGGAAGCGCATG
**bJun-NheI-r**	CAGgctagcGGTGGCGATGGATCTTCTAG
**NheI-RSPC**	ACCgctagcCTGGTAAGCAAGGGCGAGG
**BamHI-bFos**	TGGggatccgccaccatggTGGGTCGTGCGCAGTCCATCGG
**bFos-NheI-r**	CATgctagcGTGGTTCATGACTTTCTG
**NheI-RSPN**	CACgctagcATGAGGAAACCGTTCCGTG
**RSPN-HindIII-r**	GATaagcttCTACTTGTACAGCGCGTCCGTG

Table S2. In vitro ΔF/F response of truncated versions (with deleted M13-like peptide) of the purified FRCaM and GCaM6s indicators to the saturating calcium ion concentrations.

Indicator	ΔF/F		
	**0-39 μM [a]**	**0-820 μM [a]**	**0-2000 μM [a]**
**FRCaM**	0.17 ± 0.03	0.00 ± 0.04	0.04 ± 0.01
**GCaM6s**	0.16 ± 0.03	0.18 ± 0.02	0.05 ± 0.03

[a] 39, 820, and 2000 μM free calcium concentration corresponds to the 30 mM MOPS, 100 mM KCl, pH 7.20 buffer supplemented with either 10 mM CaEGTA, or 10 mM CaNTA or 2 mM $CaCl_2$, respectively. 0 free calcium concentration corresponds to the 30 mM MOPS, 100 mM KCl, pH 7.20 buffer supplemented with either 10 mM EGTA, or 10 mM NTA or 0 mM $CaCl_2$, respectively. Data were averaged across 8 repeats. SD is shown.

NES-FRCaMP-stop gene:

ATGCTTCAACTTCCTCCTCTTGAACGTCTTACTCTTTCGAGATCTATGAGGAAACCGTTCCGTGGC
GCGGGCAACGCTGTGCGTGCTTTCAGCACTTGGAAAAAGCTAGTGCCGCGGGTTTCCGAGTGGAT
GTACCCCGAGGACGGCGCCCTGAAGAGCGAGATCAGGAAGGGGCTGAGGCTGAAGGACGGCGG
CCACTACGCCGCCGAGGTCAAGACCACCTACAAGGCCAAGAAGCCCGTGCAGCTGCCCGGCGC
CTACATCGTCGACATCGAGTTGGACATCTTGTCCCACAACGAGGACTACACCATCGTGGAACAGT
GCGAACGCGCCGTGGGCCGCCACCCCACCGGTGGCACGGACGCGCTGTACAAGGGAGGTACAG
GCTCCGGCGGGAGTCTGGTAAGCAAGGGCGAGGAGGATAACATGGCCATCATCAAGGAGTTCAT
GCGCTTCAAGGTGCACATGGAGGGCTCCGTGAACGGCCACGAGTTCGAGATCGAGGGCGAGGG
CGAGGGCCGCCCCTACGAGGCCTTCCAGACCGCTAGGCTGAAGGTGACCAAGGGTGGCCCCCTG
CCCTTCGCCTGGGACATCCTGTCCCCTCAGTTCATGTACGGCTCCAAGGCCTACATTAAGCACCC
AGCCGACATCCCCGACTACTTCAAGCTGTCCTTCCCCGAGGGCTTCAGGTGGGAGCGCGTGATGA
GCTTCGAGGACGGCGGCATTATTCACGTTAATCAGGACTCCTCCCTGCAGGACGGCGTATTCATC
TACAAGGTGAAGCTGCTCGGCACCAACTTCCCCCCCGACGGCCCCGTAATGCAGAAAGAGACCA
TGGGCTGGGAGGCTTCCTACGAGATGACTACCCGTAACCTTACAGATGAGCAGATTGCGGAGTTC
CGTGAGGCCTTTTCGCTGCTTGATCGTGATCAGGATGGAAATATCACGTCCAATGAATTGGGTGT
GGTTATGAGGTCGTTAGGTCTATCGCCTACTGCCGCCGAATTACAAGATATGATTAATGAGGTCG
ATGCCGATGGTAATGGCACAATTGATTTTACCGAATTTTTGACAATGATGGCCCGGAAAATGAAG
GATACCGACGACGAAGAGGAAGTTCGCGAAGCCTTTAAAGTCTTCGATAAAGATGGAAGTGGAT
ACATTACAGTCGAGGAGCTGACTCATGTTCTTACAAGTCTCGGTGAACGTTTGTCTCGAGAAGAA
GTAGCCGATGTGATACGTGAAGCCGACTCCGATGGCGATGGTGTAATCAACTACGAAGAATTCT
CTCGTGTCATTTCTTCCAAGTAA

Figure S1. Nucleotide sequence of NES-FRCaMP protein.

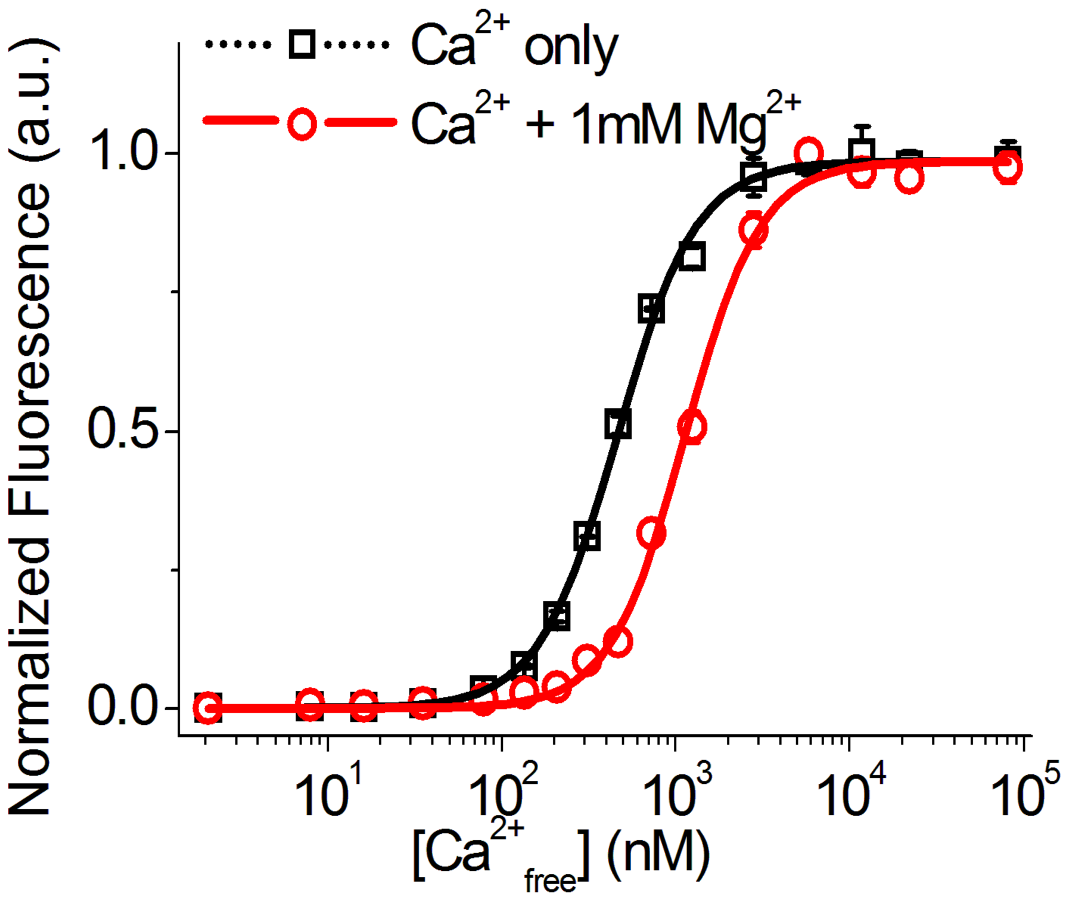

Figure S2. Calcium titration curves for R-GECO1.

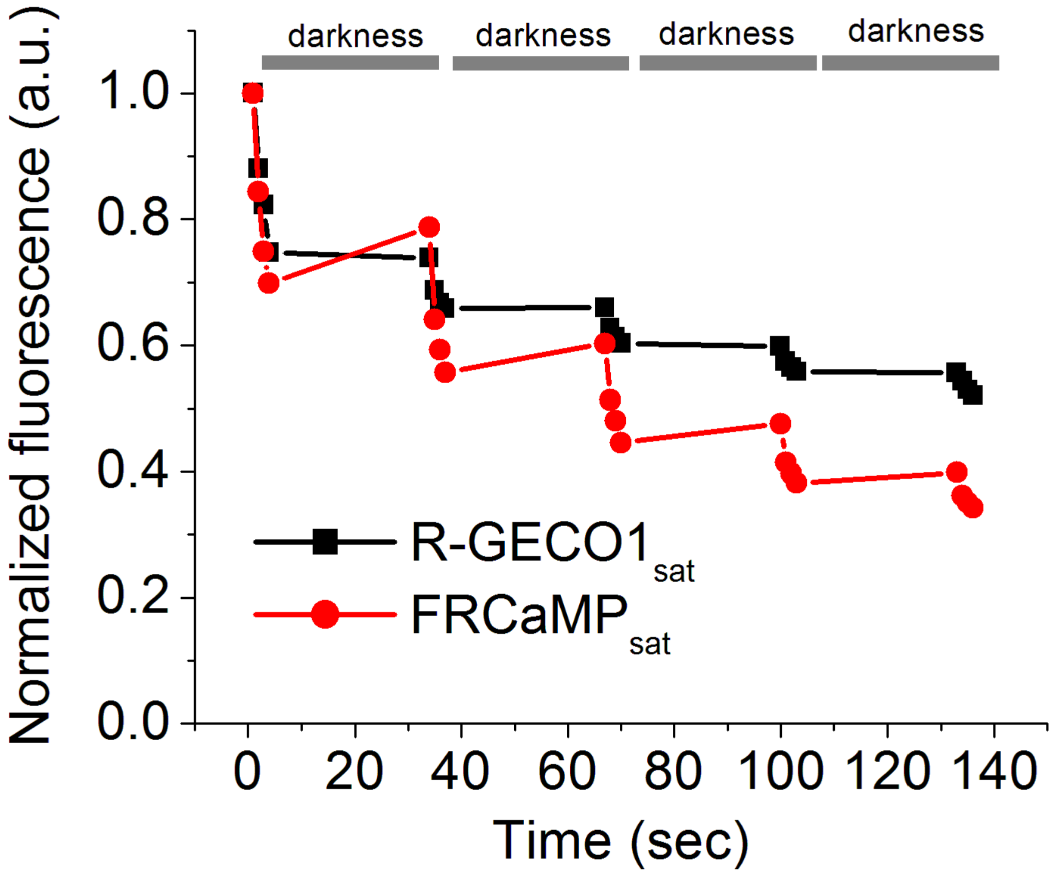

Figure S3. Photochromism in the FRCaMPsat indicator. 5 cycles of continuous metal halide lamp illumination were composed of photobleaching with yellow light (550/25BP) for 4 seconds followed by 30 seconds of darkness between cycles (marked as grey lines). Data were averaged across 6 drops in oil from 3 fields of view.

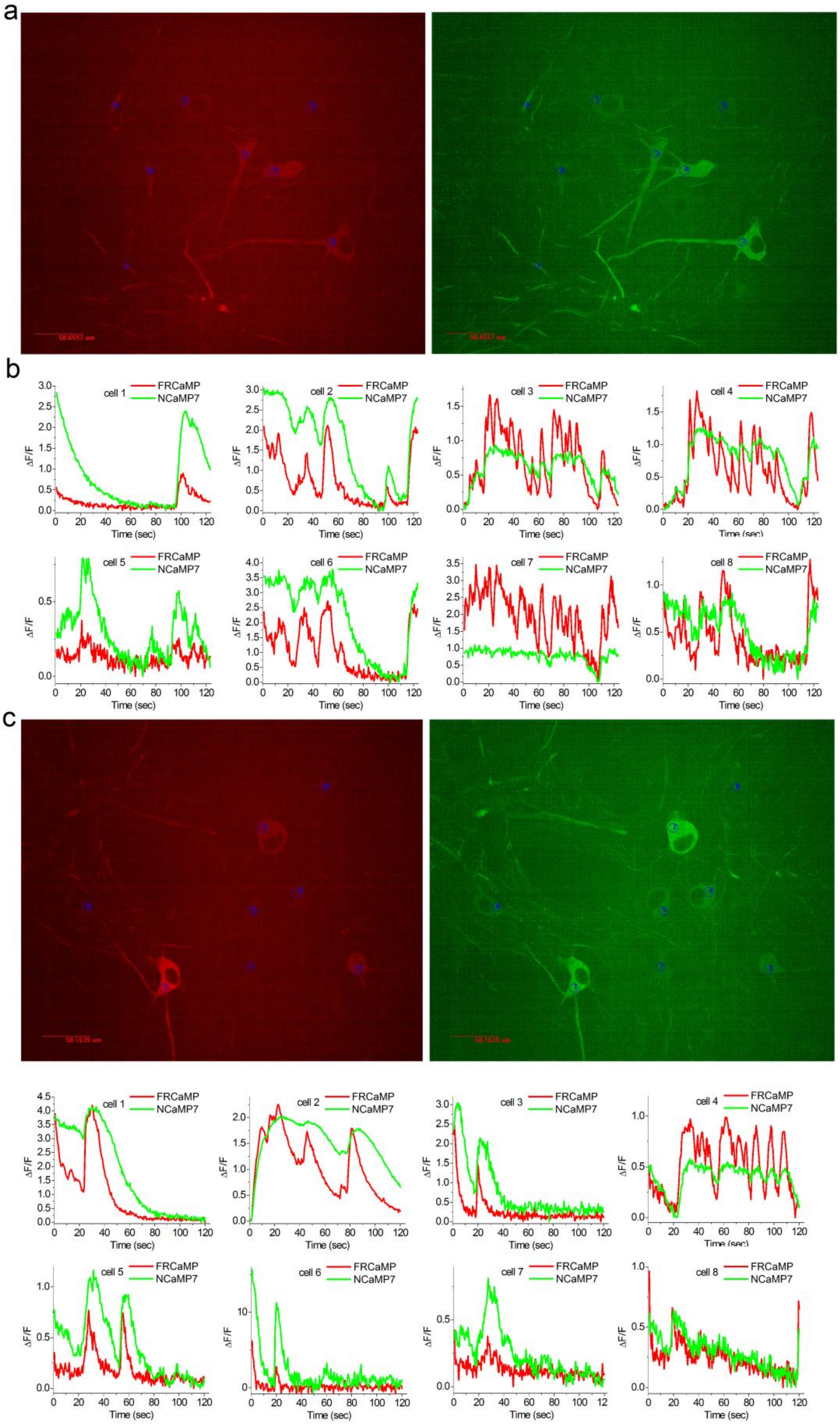

Figure S4. Calcium imaging of non-specific (spontaneous) activity of neuronal cultures co-expressing the FRCaMP and NCaMP7 calcium indicators. (**a, c**) Confocal images of two fields of view for neuronal cultures co-expressing the FRCaMP and NCaMP7 indicators. Scale bar, 50 μm. (**b, c**) Examples of ΔF/F traces for the 8 cells are shown for each of two fields of view. Neuronal cultures co-expressing the NES-FRCaMP and NES-NCaMP7 indicators were imaged on DIV 15th. Neuronal cultures were transduced on DIV 4th with the mixture of rAAVs carrying NES-FRCaMP and NES-NCaMP7.

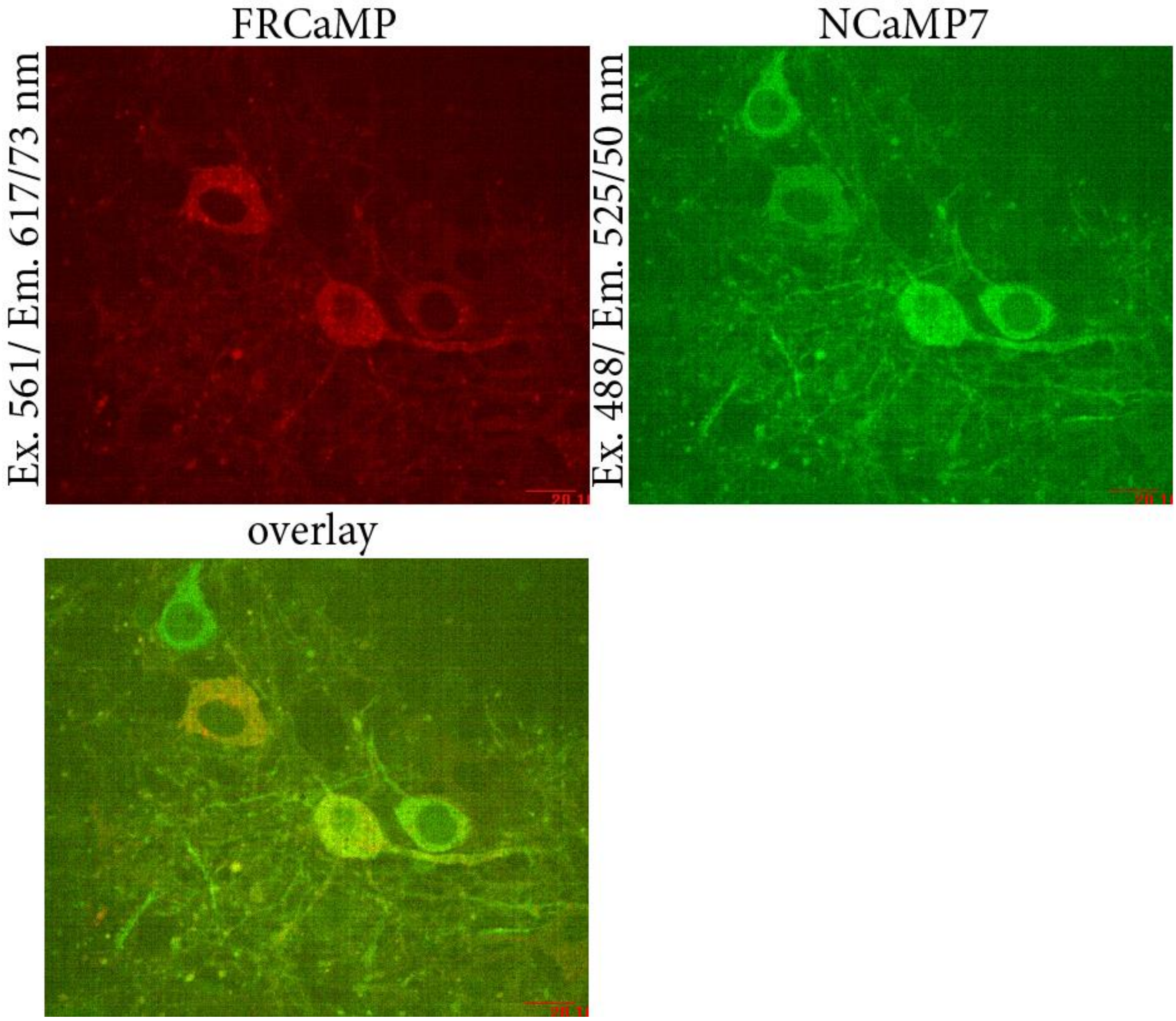

Figure S5. Puncta-like localization of the red FRCaMP indicator co-expressing with green NCaMP7 in cultured neurons. Confocal images of neurons co-expressing green NCaMP7 indicator with even distribution and red indicator FRCaMP with uneven puncta-like distribution. Neuronal cultures were imaged on DIV 22th. Scale bar, 20 μm.

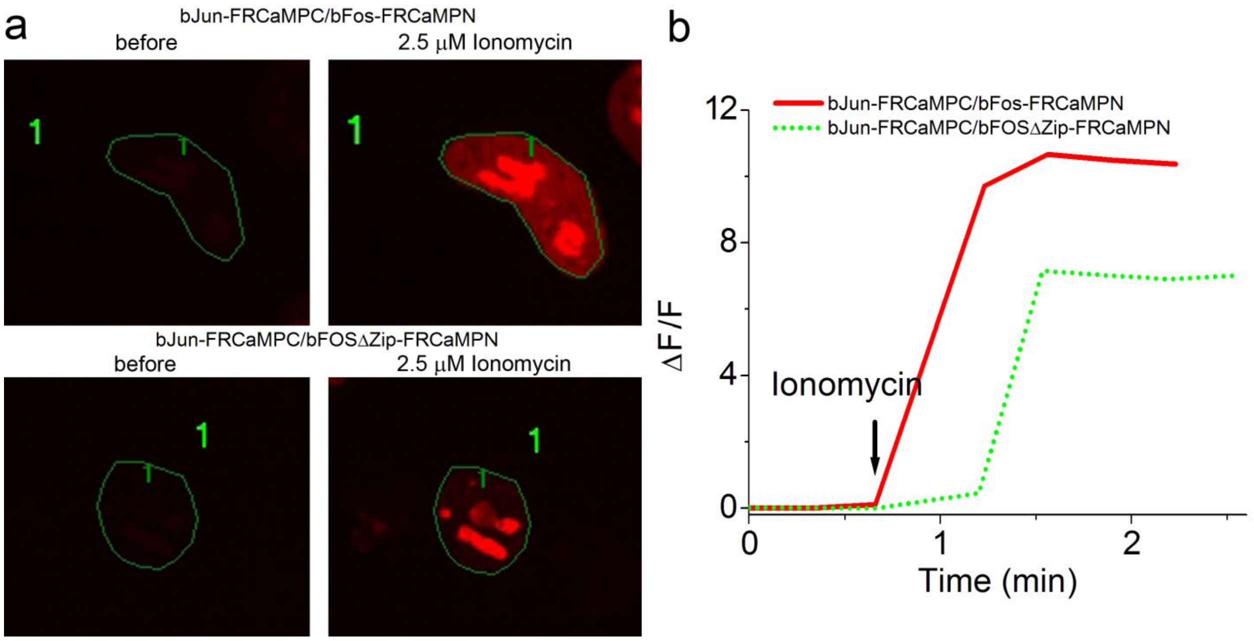

Figure S6. Localization and time-lapse response of the split-version of the FRCaMP indicator to Ca^{2+} variations in HeLa cells depending on the presence of the heterodimerizing (bJun-FRCaMPC/bFos-FRCaMPN) or non-heterodimerizing (bJun-FRCaMPC/bFOSΔZip-FRCaMPN) pair. **(a)** Zoomed area of the cell selected on Figure 7a. **(b)** The graph illustrates ΔF/F changes over time in red fluorescence of the split-version of the FRCaMP indicator in response to the addition of 2.5 μM of ionomycin depending on the presence of the heterodimerizing (bJun-FRCaMPC/bFos-FRCaMPN) or non-heterodimerizing (bJun-FRCaMPC/bFOSΔZip-FRCaMPN) pair. The changes on the graph correspond to the area indicated, as a numbered circle on the panel a, Figure 7 or panel a, Figure S5. Time of ionomycin addition is shown by arrow.

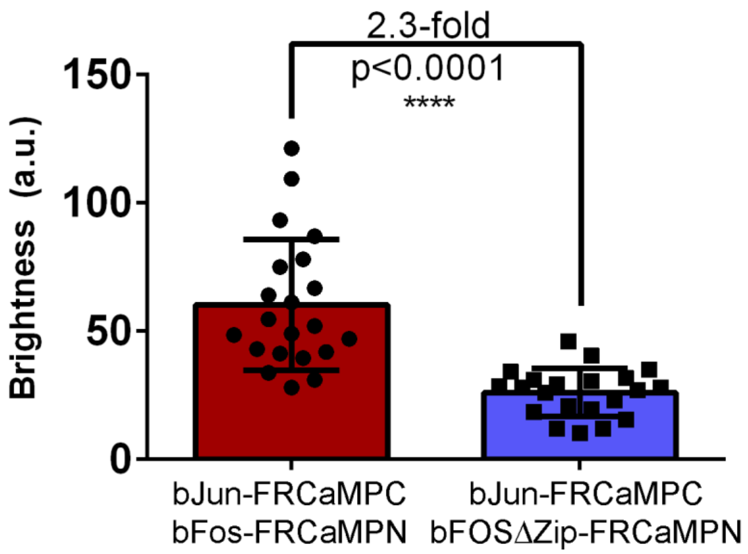

Figure S7. Brightness of the split-version of the FRCaMP indicator at low Ca^{2+} concentrations in HeLa cells depending on the the presence of the heterodimerizing (bJun-FRCaMPC / bFos-FRCaMPN) or non-heterodimerizing (bJun-FRCaMPC / bFOSΔZip-FRCaMPN) pair. Comparison of the averaged brightness (for the heterodimerizing bJun-FRCaMPC and bFos-FRCaMPN split calcium indicator and its control none-heterodimerizing bJun-FRCaMPC and bFos-FRCaMPN split calcium indicator in HeLa cells at physiological calcium concentration before ionomycin addition. Error bars are standard deviations across twenty one cells (three cultures). *p* values show statistical difference between the respective values. ****, *p* - value is lower than 0.0001.
